# Parallelized Dilate Algorithm for Remote Sensing Image

**DOI:** 10.1155/2014/286963

**Published:** 2014-05-11

**Authors:** Suli Zhang, Haoran Hu, Xin Pan

**Affiliations:** ^1^School of Computer Project & Technology, Changchun Institute of Technology, Changchun 130012, China; ^2^School of Computer & Information, Anqing Teachers College, Anqing 246011, China

## Abstract

As an important algorithm, dilate algorithm can give us more connective view of a remote sensing image which has broken lines or objects. However, with the technological progress of satellite sensor, the resolution of remote sensing image has been increasing and its data quantities become very large. This would lead to the decrease of algorithm running speed or cannot obtain a result in limited memory or time. To solve this problem, our research proposed a parallelized dilate algorithm for remote sensing Image based on MPI and MP. Experiments show that our method runs faster than traditional single-process algorithm.

## 1. Introduction

Land use/cover information has been identified as one of the crucial data components for many aspects of global change studies and environmental applications. The development of remote sensing technology has increasingly facilitated the acquisition of such information [1]. As an important algorithm, dilate algorithm can give us more connective view of a remote sensing image which has broken lines or objects. However, with the technological progress of satellite sensor, the resolution of remote sensing image has been increasing and its data quantities become very big. This would lead to the decrease of algorithm running speed or cannot obtain a result in limited memory or time.

Paralleled program can split a big computing task into subcomputing tasks and make full use of the advantage of multicore and multicomputer to improve the computing speed [2]. To accelerate the process speed of remote sensing images algorithm, many methods had been proposed: parallel k-means or EM cluster method for remote sensing image [3, 4]. Wang utilized loud computing to a rapid processing of remote sensing images [5]. Parallel classification method has been proposed to archive a faster remote sensing images training speed [6, 7]. Parallel program can be further divided into multiprocesses parallel and multithreads parallel. Message passing interface (MPI) is a library specification for message passing, proposed as a standard by a broadly based committee of vendors, implementers, and users [8]; we can realize multiprocesses. Multiprocessing (MP) is the use of two or more central processing units (CPUs) within a single computer system [9].

In this research, we introduce MPICH2 and OpenMP technology and propose a parallelized dilate algorithm for remote sensing image (PDARSI); through PDARSI a big dilate task can be split into a lot of subtasks; each subtask can run on corresponding computer or core. Experiments show that our method runs obviously faster than traditional single-process algorithm.

## 2. Preliminary Knowledge

### 2.1. Dilate Algorithms

There are two sets *A* and *B* in *Z*; a complement set of *A* is as follows:

(1) $$\begin{array}{c} A^{C}=\left\{\omega \;|\;\omega \notin A\right\}. \end{array}$$

Based on this formula the difference of *A* and *B* represented by *A* − *B* can be defined as

(2) $$\begin{array}{c} A-B=\left\{\omega \;|\;\omega \in A,\omega \notin B\right\}=A\cap B^{C}. \end{array}$$

The reflection of *B* represented as $\hat{B}$ can be defined as

(3) B^={ω ∣ ω=−b,b∈B}.

The set *A* move to point *z* = (*z*1, *z*2)'s location represented by (*A*)_*z*_ can be defined as

(4) $$\begin{array}{c} {\left(A\right)}_{Z}=\left\{c\;|\;c=a+z,a\in A\right\}. \end{array}$$

The binary dilation of *A* by *B*, denoted by *A* ⊕ *B*, is defined as the set operation:

(5) $$\begin{array}{c} A⊕B=\left\{z\;|\;{\left(\hat{B}\right)}_{z}\cap A\neq \phi \right\}. \end{array}$$

Here B^ is the reflection of the structuring element *B*. In other words, it is the set of pixel locations *z*, where the reflected structuring element overlaps with foreground pixels in *A* when translated to *z*. Note that some people use a definition of dilation in which the structuring element is not reflected [10]. In the general form of gray-scale dilation, the structuring element has a height. The gray-scale dilation of *A*(*x*, *y*) by *B*(*x*, *y*) is defined as

(6) (A⊕B)(x,y) =max⁡{A(x−x′,y−y′)+B(x′,y′) ∣ (x′,y′)∈DB},

where *D*_*B*_ is the domain of the structuring element *B* and *A*(*x*, *y*) is assumed to be −*∞* outside the domain of the image. To create a structuring element with nonzero height values, use the syntax strel (nhood, height), where height gives the height values and nhood corresponds to the structuring element domain, *D*_*B*_ [11].

### 2.2. MPI and OpenMP

Message passing interface (MPI) is a standardized and portable message-passing system designed by a group of researchers from academia and industry to function on a wide variety of parallel computers. OpenMP is a portable, scalable model that gives shared-memory parallel programmers a simple and flexible interface for developing parallel applications for platforms ranging from the desktop to the supercomputer. We can use MPI in the cluster computers to realize multiprocess parallelization, and each process adopts OpenMP to realize multithreading parallelization.

## 3. Parallelized Dilate Algorithm for Remote Sensing Image

**Figure 1:**
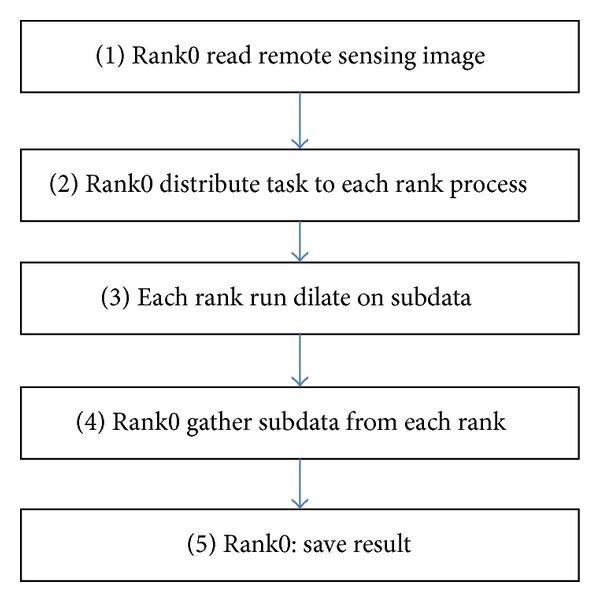
Five stages of algorithms.

The generic process of parallelized dilate algorithm for remote sensing image (PDARSI) is shown in Figure 1.

Firstly, in main function, MPI interface start and initial all the processes by:

 MPI::Init(argc, argv); //Initial all the process;
 
*size = MPI::COMM_WORLD.Get_size(); *//Get number of processes;
 
*rank = MPI::COMM_WORLD.Get_rank(); *//Get current process's rank number.

And then the algorithm is divided into five steps: (1) Rank0 read the entire remote sensing data, and data in accordance with the number of processes is divided into multiple subdata; (2) Rank0 send data, and the data is distributed to each process; (3) each rank processes its own data to obtain the corresponding results; (4) Rank0 collect all the data, and data integration as a result; (5) Rank0 write the result to disk. Finally, in main function, call

 
*MPI::Finalize()*; //stop all the process.

All the process was destroyed and the algorithm was ended.

*Stage 1.* Reading stage: in order to solve the problem of image data read, task assignment, and data transmission, PDARSI adopt a Plines class to store the remote sensing data; Plines class has the following functions: (1) storing remote sensing image in units of row; (2) the storage part of the data; (3) redundant storage of data boundary information; (4) supporting serialization; and (5) supporting the reconstruction of the data; the process of Rank0's reading and splitting can be described in Algorithm 1.

Through this procedure, Rank0 can split all the data into subdata corresponding to each Rand, and the PDARSI proceed to Step 2. In Step 2, Rank0 send all the subdata to Rank0 to Rankn by

 
*MPI::COMM_WORLD.Send(&linehead,sizeof(LineHead),MPI::BYTE,i,0); *//send Plines head
 
*MPI::COMM_WORLD.Send(lines[i].GetSendBufferHead(),(int)linehead.sendsize,MPI::BYTE,i,0); *//send PLines data.

**Figure 2:**
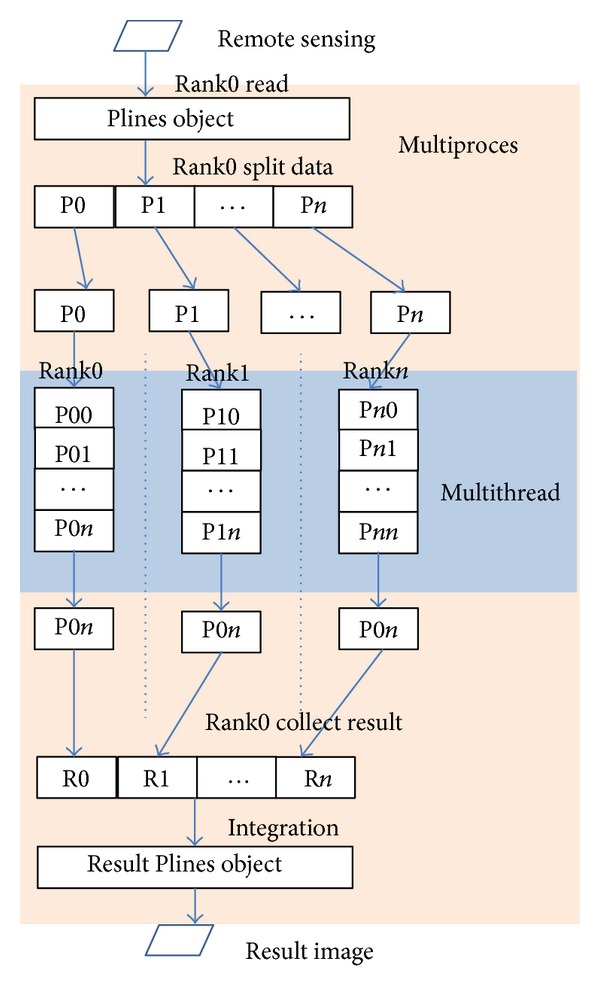
Plines object and its status at different stage.

In Step 3, Plines object which own by each rank was further split into Plines array and each object of array run dilate algorithm and obtain result in a thread. In Step 4, Rank0 collect all the results from every rank process and integrate them as a result. In Step 5, Rank0 save the result to a disk. The Plines objects send and receive figure can be seen in Figure 2.

Through PDARSI remote sensing image can dilate parallel in multiprocess and multithread.

## 4. Experiments

**Figure 3:**
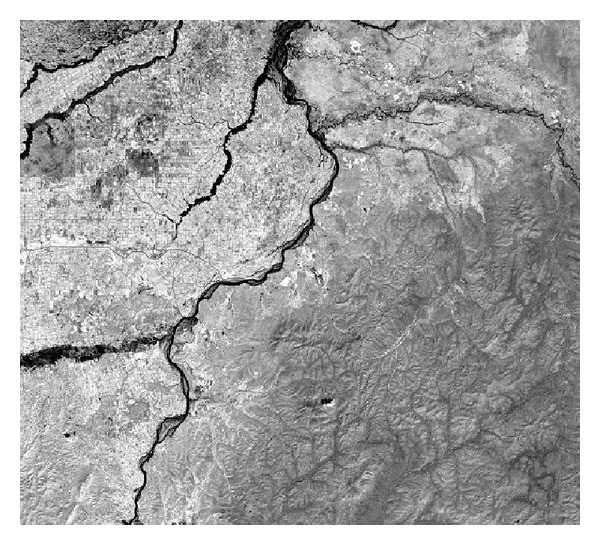
Test remote sensing image.

This research chooses Landsat-5 TM image and extracts a band for test image; the image size is 5230 × 4736 and 23.6 M (see Figure 3).

To test the efficiency of PDARSI algorithm, we adopt a HPC cluster which contains Intel i5 2300 computer as head node; it controls two compute nodes which have AMD FX8350 8-core CPU. Each computer of cluster utilizes Fedora 16 64-bit Linux operating system and MPICH2 as MPI management interface and OpenMP as multithread library. In order to test the effectiveness of parallel algorithms, we adopt the number of processes from 1 to 10 and the number of threads from 1 to 10, totally 100 times test. The dilate operator use 15 × 15, in each pixel of image algorithm would compute 15 × 15 = 225 times, Table 1 is test result.

It can be seen from Table 1, when there are one processes and one threads, the algorithm is equivalent to the traditional single-process algorithm, the running time is slowest 79.03 seconds, with the number of processes or the number of threads increase the running speed become faster. when 10 processes and one thread the algorithm running time is 22.67 seconds, only 0.27 times of the single-process run time. When 10 threads and one process the running time is 15.8 seconds, only 0.2 times of the single-process run time. Both MP and MPI can play an important role in accelerating the program running speed.

**Figure 4:**
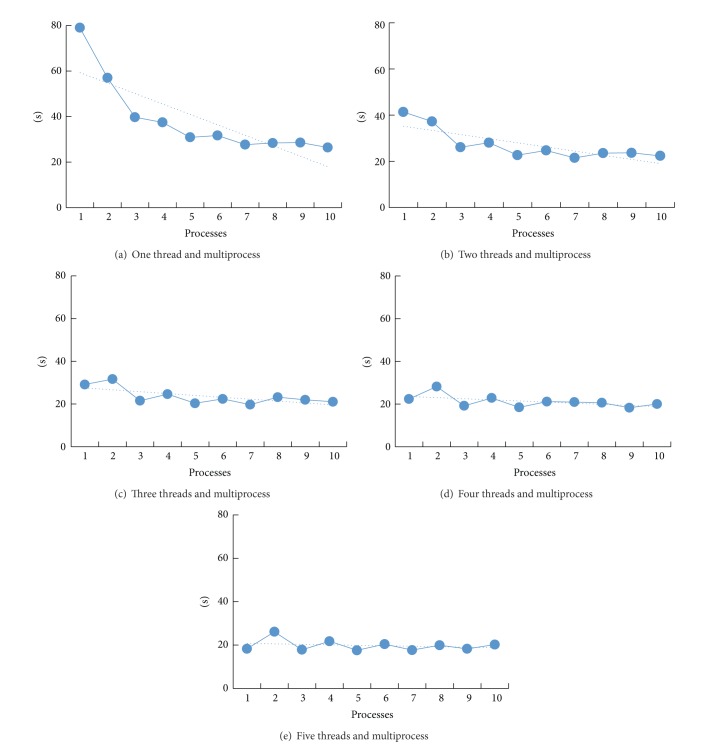
Algorithm speed and its relation to the number of processes and 1–5 threads.

Figure 4 is algorithm speed and its relation to the number of processes and 1–5 threads.

**Figure 5:**
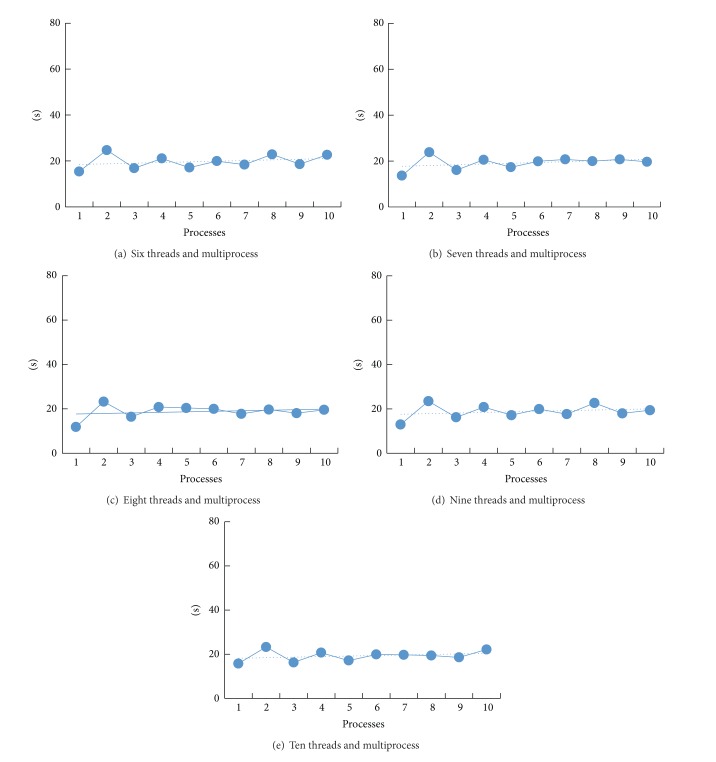
Algorithm speed and its relation to the number of processes and 6–10 threads.

As can be seen from Figure 4(a), the elapsed time of algorithm declined along with the increasing number of processes, but the trend became slower when the processes number exceeds 4. Figures 4(b) and 4(c) show that the algorithm's speed increases more slowly when the thread process number is bigger than 1; this means that threads can bring more increase than the processes. From Figures 4(d) and 4(e), the number of threads in the initial stage more than 4, number of processes' increase may actually reduce the operating speed, which is due to the improvement of the process of the speed has less influence than the speed of data transmission between processes.  Figure 5 is algorithm speed and its relation to the number of processes and 6–10 threads.

In Figure 5, there are less differences among the five figures, due to HPC cluster computer hardware limitations (the compute nodes containing a total of 16 cores) and the time-consuming communication among processes. The algorithm's speed is not linear with the number of processes and threads, and when the speed limitation is reached, the increase of the number of processes or threads will not increase running speed or even decrease the speed. The algorithm will archive better result when more powerful HPC cluster is utilized.

**Figure 6:**
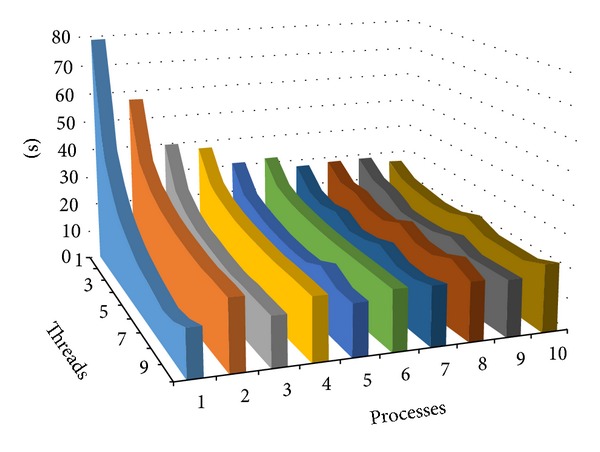
The relation between threads and processes.

The relation between threads and processes can be seen from Figure 6. Multithread method does not require data transmission so is can bring more obvious increase in algorithm speed, since algorithms require the transmission of data between two computers in multiprocess stage, when process number is even algorithm need transmitted half of the data from Rank0 node to another node, so the speed is more faster in processed number is odd.

**Figure 7:**
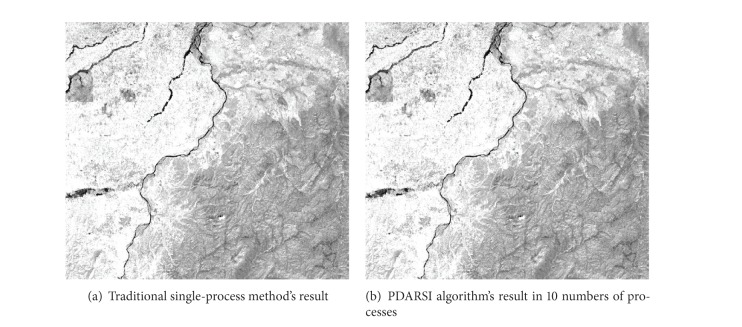
Result comparison.

The results of the PDARSI algorithm and the traditional single-process algorithm can be seen in Figure 7.

PDARSI algorithm splits a remote sensing image into subdata, and each subdata has* UpperBuffer* and* BottomBuffer* to ensure a pixel which at subdata border can access neighbor pixels on the other subdata; this mechanism guarantees that the dilation algorithm can obtain right result even the whole calculate task are partitioned into processes. When the dilation algorithm calculation in each process is completed, Rank0 collect the results from processes and integrate all the results into a result image. As can be seen from Figure 7, PDARSI does not change the results of the original algorithm and result images are exactly the same; this proves that our proposed algorithm can accelerate running speed and does not alter the results of the original algorithm.

## 5. Conclusions

This research uses MPICH2 and OpenMP to design a parallelized dilate algorithm; it can take full advantage of HPC cluster computing resources and achieve the purpose of rapid processing of remote sensing image. Through PDARSI a big dilate task can be split into a lot of subtasks; each subtask can run on corresponding computer or core. Experiments show that our method runs obviously faster than traditional single-process algorithm.

## Figures and Tables

**Algorithm 1:**
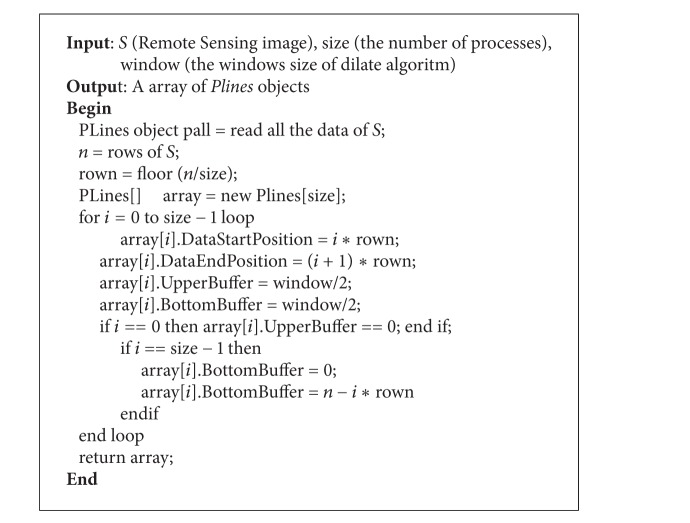


**Table 1:** Run speed in multiprocess and multithread.

The number of processes	The number of threads									
	1	2	3	4	5	6	7	8	9	10
1	79.03	41.36	29.16	22.35	18.27	15.38	13.63	11.84	13	15.8
2	57.02	37.27	31.63	28.19	26.1	24.73	23.87	23.24	23.5	23.29
3	39.74	26.11	21.54	19.15	17.82	16.83	16.05	16.42	16.21	16.3
4	37.46	28.08	24.64	22.85	21.78	21.08	20.55	20.81	20.8	20.68
5	30.88	22.7	20.36	18.47	17.58	17.1	17.29	20.42	17.2	17.14
6	31.69	24.73	22.38	21.14	20.42	19.93	19.89	19.98	19.9	19.88
7	27.67	21.49	19.71	20.92	17.71	18.42	20.71	17.75	17.62	19.74
8	28.39	23.56	23.22	20.61	19.93	22.9	19.96	19.69	22.66	19.45
9	28.59	23.7	21.98	18.31	18.24	18.62	20.69	18.02	18.02	18.56
10	26.43	22.35	21.08	20.01	20.25	22.67	19.67	19.55	19.41	22.15
